# Treatment of older patients with Hodgkin lymphoma

**DOI:** 10.1007/s44313-025-00084-4

**Published:** 2025-06-12

**Authors:** Chung Hyun Park, Hyunsoo Cho, Soo-Jeong Kim

**Affiliations:** 1 Blood Cancer Research Institute, Yonsei University College of Medicine, Seoul, 03722 Republic of Korea; 2 Division of Hematology and Oncology, Department of Internal Medicine, Yongin Severance Hospital, Yongin-si, Gyeonggi-do 16995 Republic of Korea; 3 Division of Hematology, Department of Internal Medicine, Yonsei University College of Medicine, Seoul, 03722 Republic of Korea

**Keywords:** Older patients, Frailty, Geriatric assessment, Hodgkin lymphoma

## Abstract

Older patients with classic Hodgkin lymphoma (HL) often experience poor outcomes due to age-related comorbidities and treatment-related toxicity. Comprehensive geriatric assessment and supportive care measures, including pre-phase corticosteroids, growth factor prophylaxis, and organ function monitoring, are essential for optimizing treatment tolerance in this vulnerable patient population. Recent phase III trial (S1826) demonstrated that nivolumab plus doxorubicin, vinblastine, and dacarbazine (Nivo + AVD) significantly improves progression-free survival and is better tolerated than brentuximab vedotin (BV) + AVD, particularly in patients over 60 years of age. Given its efficacy and reduced toxicity, Nivo + AVD is likely to become a key treatment option for fit older patients with HL. For frail patients, chemo-free approaches with BV and checkpoint inhibitors remain viable alternatives. Future research should refine fitness-based treatment strategies, integrate novel agents, and enhance supportive care to improve outcomes and minimize treatment-related toxicity in this population.

**Graphical Abstract:**

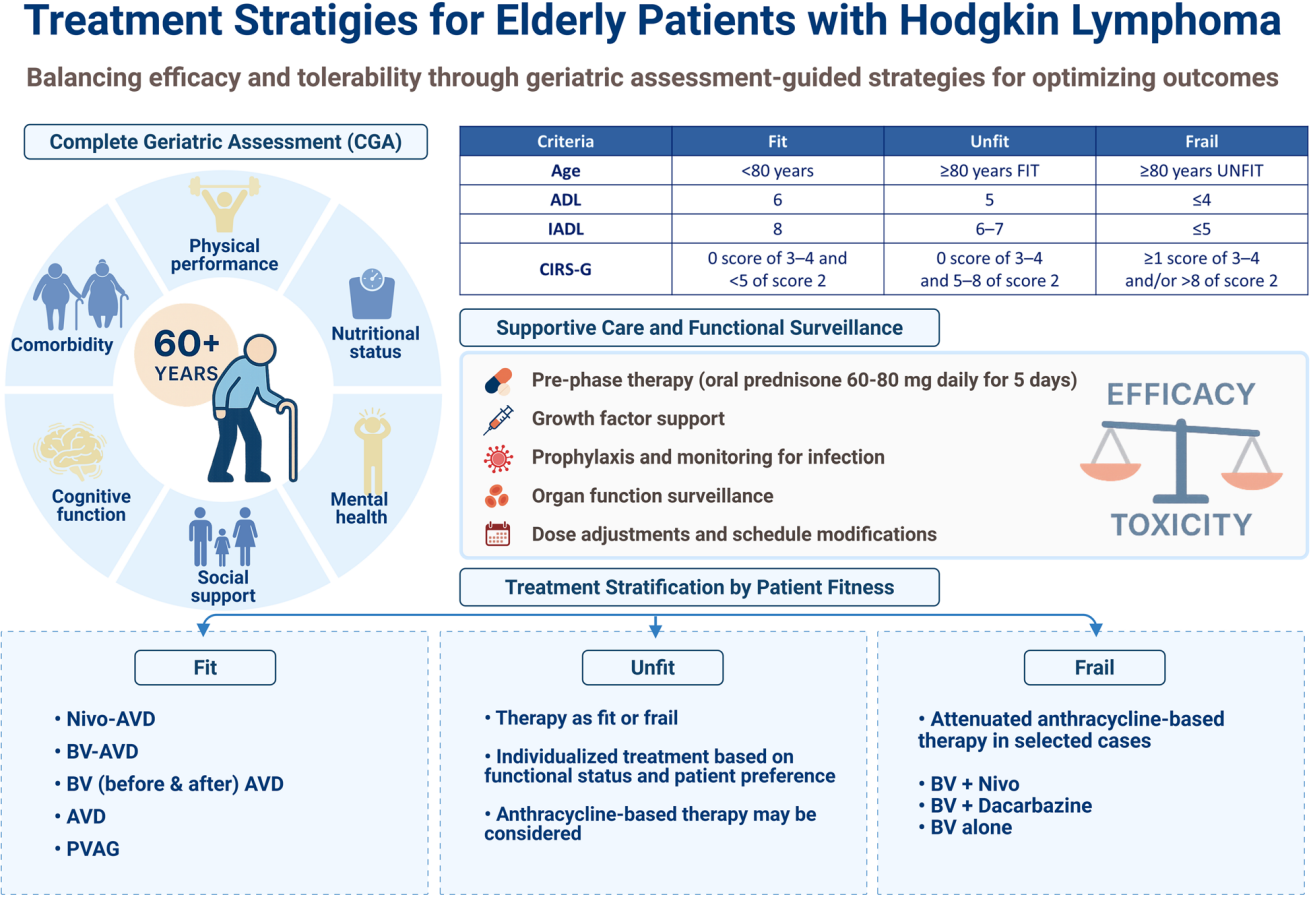

## Introduction

### Importance of advancing treatment strategies for older patients with hodgkin lymphoma

Hodgkin lymphoma (HL) has a bimodal age distribution, with a second incidence peak in later adulthood, affecting 20–25% of patients aged > 60 years [1]. While classic Hodgkin lymphoma (cHL) is generally regarded as a curable disease, long-term outcomes in older adults remain substantially inferior, with 5-year overall survival (OS) rates ranging from 49 to 65%, compared to approximately 90% in younger patients [2–4]. In real-world clinical practice, older patients exhibit higher relapse rates and increased treatment-related mortality, often due to chemotherapy intolerance, infections, and hematologic toxicity, which frequently lead to dose reductions or early treatment discontinuation [5]. Several additional factors contribute to poorer outcomes in older HL. Biologically, higher prevalence of mixed cellularity, lymphocyte-depleted HL and Epstein Barr virus (EBV)-associated disease in older HL may confer a more aggressive course [6]. Comorbidities and functional decline in the elderly may reduce chemotherapy tolerance due to organ dysfunction and frailty [7]. Therefore, standard chemotherapy is poorly tolerated primarily due to bleomycin-induced pulmonary toxicity and anthracycline-related cardiotoxicity, and intensive regimens are generally infeasible [8].

Given these challenges, there is a critical need for tailored treatment strategies that improve outcomes while minimizing toxicity in older HL patients. As the elderly population continues to grow and the limitations of standard therapy become more evident, efforts have intensified to develop age-adapted treatment approaches that balance efficacy and safety. This requires a multifaceted approach: (1) employing geriatric assessment to individualize treatment intensity; (2) optimizing supportive care to reduce treatment-related complications; (3) incorporating targeted agents (antibody–drug conjugate and PD-1 checkpoint inhibitors) which may be alternatives or adjuncts to chemotherapy​; and (4) refining conventional chemotherapy regimens (dose attenuations or substitutions) to improve tolerability. Therefore, this review explores the latest evidence and expert recommendations on treating older HL patients, covering geriatric assessment for risk stratification, supportive care strategies including pre-phase therapy, conventional chemotherapy approaches and modifications, emerging targeted therapies in frontline treatment, and future directions.

### Rationale for Geriatric Assessment (GA) and its impact on treatment

Chronological age alone is a crude indicator of a patient’s fitness for therapy. We now recognize that older patients are a heterogeneous group, as some fit seniors can tolerate intensive treatment, whereas frail ones cannot, despite similar ages [9]. Although Eastern Cooperative Oncology Group (ECOG) Performance Status (PS) and Karnofsky Performance Status (KPS) are widely used to assess the degree of a patient’s functional impairment, they do not fully capture the physiological reserve and vulnerabilities of older patients​. Comprehensive GA (CGA) is an interdisciplinary evaluation of medical, functional, cognitive, and psychosocial domains that provides a more nuanced assessment of an older patient’s health status [9]. CGA can uncover occult issues (e.g. subtle cognitive impairment, poor nutrition, or unaddressed comorbidities) and stratify patients by fitness level, guiding treatment tailoring to avoid both under-treatment and over-treatment​. In older patients, performing a CGA prior to therapy is increasingly recognized as indispensable for informing therapy decisions and predicting tolerance [10].

Multiple GA tools and scoring systems have been developed and studied in lymphoma including HL. These include general oncology GA tools such as the G8 screening tool, Charlson Comorbidity Index (CCI), Cumulative Illness Rating Scale for Geriatrics (CIRS-G), and scales assessing activities of daily living (ADL) and instrumental ADL (IADL), as well as lymphoma-specific adaptations. For example, CIRS-G quantifies comorbidity burden; a simplified GA can classify patients as fit, unfit, or frail in aggressive lymphoma [11]. Geriatric assessment results have prognostic implications in older HL. In a multicenter trial where patients ≥ 60 received sequential brentuximab vedotin (BV) and doxorubicin, vinblastine, and dacarbazine (AVD) chemotherapy, baseline GA findings stratified outcomes dramatically: patients with low comorbidity (CIRS-G < 10) had a 2-year progression-free survival (PFS) of 100%, versus only 45% if CIRS-G ≥ 10 [12]. Likewise, those with no impairment in instrumental ADLs had a 2-year PFS of 94%, compared with 25% if any IADL dependence was present [12]. These data underscore that frailty as measured by GA correlates with a markedly higher risk of treatment failure. Similarly, a retrospective study found that older HL patients with severe comorbidities (CIRS grade ≥ 3) had 3-year OS of only ~ 46% versus 88% in those without severe comorbid illness [7]. Notably, even among patients deemed fit enough for standard chemotherapy, those with high comorbidity still fared worse, indicating that frailty exerts an independent adverse effect despite aggressive therapy​.

### Integration of GA into treatment planning

Because GA identifies vulnerabilities that might not be evident from routine evaluation, it directly informs the choice and intensity of therapy. Fit older patients (GA indicating robust functional status and controlled comorbidities) are often treated similarly to younger patients, with curative intent regimens, whereas frail patients (GA revealing significant deficits) may need dose-reduced or alternative regimens​ [13]. For intermediate or unfit patients, GA can highlight specific issues to address. For instance, borderline cardiac function might prompt use of a liposomal doxorubicin or cardioprotective strategies rather than excluding anthracycline altogether [14]. GA results should ideally be used to categorize patients (fit, unfit, or frail) in a standardized way; however, clear consensus on categorization in HL is still evolving. Most existing classifications for lymphoma fitness (e.g. the Italian Lymphoma Group’s simplified GA) were developed in diffuse large B-cell lymphoma (DLBCL) and are retrospectively applied to HL​ [11]. Prospective HL-specific GA studies are needed to refine these tools for this disease. Nonetheless, current expert guidelines strongly recommend performing an objective GA in all older HL patients​ [15].

In practice, a CGA for an older HL patient typically evaluates: comorbid conditions (e.g. cardiovascular, pulmonary diseases), medications, physical function (ADL/IADL, gait speed or get up and go test), nutrition (e.g. weight loss, serum albumin), cognitive status, psychological state, and social support. Deficits in these domains can be targeted with interventions before or during therapy. For example, if GA finds poor mobility or deconditioning, physical therapy can be initiated; if a patient has borderline diabetes control or chronic obstructive pulmonary disease, these can be optimized prior to chemotherapy [16]. Such interventions can expand the pool of fit patients by mitigating risk factors. However, even fit older patients are biologically more vulnerable than younger patients, as age-related reductions in marrow reserve and organ function mean higher risk of cytopenias and neurotoxicity​ [17]. Therefore, even robust older patients benefit from close monitoring and supportive care. GA is prognostic not only for survival but also for treatment completion, as poor GA scores predict chemotherapy modifications, early discontinuation, or hospitalization. Identifying these risks enables upfront interventions, such as growth factor support and frequent toxicity monitoring. GA can also guide treatment pathways: frail patients may receive lower-intensity regimens or novel agents in clinical trials, while fit patients undergo full-dose standard or intensified therapy. As prospective trials increasingly incorporate GA metrics, refining these tools will be crucial for improving outcomes in older HL patients.

### Conventional chemotherapy approaches

Combination chemotherapy remains the backbone of curative treatment in HL. In older patients, the challenge is to deliver an effective multidrug regimen while managing the higher risk of toxicity. Historically, the ABVD regimen (doxorubicin, bleomycin, vinblastine, dacarbazine) has been the standard first-line therapy for HL across all adult age groups [18]. However, older patients tolerate ABVD poorly due to bleomycin- and anthracycline-related toxicity [18]. Treatment-related mortality is substantially higher in the elderly receiving ABVD. For instance, among HL patients > 60 years treated on an E2496 trial, 24% developed bleomycin lung toxicity and 18% died from treatment-related causes [19]​. The risk of life-threatening pulmonary toxicity increases with age: bleomycin lung toxicity occurred in 13% of patients aged 60–69 and 24–25% of those ≥ 70, versus < 5% in patients < 50 [20]​. Moreover, the concomitant use of granulocyte colony-stimulating factor (G-CSF) with ABVD has been reported to be associated with an increased risk of bleomycin lung toxicity​ [21]. Therefore, bleomycin should be used with caution in most older HL patients, as evidenced by treatment strategies that either omit bleomycin entirely (using an AVD regimen) or limit its use to the first two cycles in older patients.

Anthracyclines, particularly doxorubicin, are essential for HL therapy as their exclusion significantly reduces cure rates [22]​. Therefore, older patients with adequate cardiac function should receive an anthracycline-based regimens. For example, adding doxorubicin (and bleomycin) to a ChlVPP regimen increased 5-year OS from 30 to 67% [23]​. Anthracycline omission should be limited to patients with absolute contraindications, such as uncontrolled heart failure. When standard doxorubicin poses a risk, alternatives like pegylated liposomal doxorubicin [24]​ or pre-treatment with the cardioprotective agent dexrazoxane [25]​ may be used.

### Outcome of conventional therapy in older HL

Despite modifications, chemotherapy alone yields lower cure rates in older HL. In fit elderly patients, 5-year OS is 50–70% for advanced-stage disease and 70–80% for early-stage disease with combined modality therapy. Survival improvements over time likely reflect better supportive care and the introduction of BV in relapse settings, with recent frontline integration of BV potentially enabling effective, less toxic regimens. In contrast, frail elderly patients ineligible for intensive therapy face poor outcomes (median survival ~ 1 year without salvage treatment), underscoring the need for novel first-line or early salvage strategies.

No single chemotherapy regimen is ideal for all older HL patients. Fit patients may receive ABVD or BV + AVD, replacing bleomycin with BV. Those with borderline fitness may benefit from sequential BV and AVD or PVAG (prednisone, vinblastine, doxorubicin, and gemcitabine) in specialized settings. Treatment should be personalized, incorporating anthracyclines when feasible and adjusting regimens (e.g., omitting bleomycin, adding growth factors) based on organ function and geriatric assessment. The emergence of targeted therapies is increasingly bridging conventional chemotherapy with novel agents in frontline treatment.

### Balancing efficacy and tolerability: the role of low-intensity chemotherapy

Low-intensity chemotherapy regimens have been developed for alternatives for older HL patients who cannot tolerate standard protocols. These regimens aim to maintain reasonable disease control while minimizing treatment-related toxicity, which is a key concern in this age group. The PVAG regimen demonstrates that carefully selected low-intensity regimens can offer durable remissions with improved tolerability in the elderly population.

- PVAG (Prednisone, Vinblastine, Doxorubicin, and Gemcitabine)
  The German Hodgkin Study Group (GHSG) introduced the PVAG regimen—prednisone, vinblastine, doxorubicin, and gemcitabine—as a frontline option for older patients with early unfavorable or advanced-stage HL [26]. In a phase II multicenter trial, 59 patients aged 60–75 years (median 68) received 6–8 cycles of PVAG. Of these, 93% had advanced-stage disease. PVAG achieved a complete remission (CR or CRu) rate of 78%, with 3-year PFS and OS rates of 58% and 66%, respectively. The regimen was feasible: 64% completed treatment per protocol, and 79% of the evaluable patients maintained a relative dose intensity (RDI) ≥80%. Toxicity was frequent but generally manageable, with 75% of patients experiencing grade 3 or 4 adverse events—primarily leukopenia (53%) and infections (23%). Only one treatment-related death (1.7%) occurred. The omission of bleomycin and dacarbazine may have contributed to improved tolerability, while a higher cumulative anthracycline dose likely preserved efficacy. Pulmonary toxicity was reported in 7% of patients but could not be definitively attributed to a specific agent due to small numbers. However, the applicability of this regimen remains limited by the study population, which included only selected elderly patients (aged 60–75 years) with good general condition (WHO PS ≤2) and preserved organ function, without undergoing a formal geriatric assessment. Consequently, its relevance to more vulnerable or frail older patients remains uncertain. Nevertheless, PVAG appears to balance efficacy and tolerability and may be a viable option for fit older patients. Future PVAG-based trials should incorporate standardized frailty measures to guide patient selection.

### Efficacy and role of targeted agents in the treatment landscape of older HL patients

Targeted therapies now offer a more selective approach in HL with fewer systemic side effects than traditional chemotherapy. In older patients, these agents reduce reliance on toxic drugs like bleomycin, enhancing tolerability. The two main classes in classical HL are the anti-CD30 antibody–drug conjugate BV and immune checkpoint inhibitors nivolumab and pembrolizumab. Both have shown strong efficacy in relapsed or refractory (R/R) HL and are increasingly being used in frontline regimens for older adults.

### Brentuximab Vedotin (BV)

BV targets CD30 on Hodgkin Reed–Sternberg (HRS) cells by delivering monomethyl auristatin E [27]. In R/R HL, BV monotherapy achieved an overall response rate (ORR) of approximately 75%, with durable remissions observed in some patients [28]. For older patients, BV is particularly appealing due to its non-overlapping toxicity profile (primarily neuropathy) as opposed to the cardiac and pulmonary toxicities associated with conventional chemotherapy. This shift in the management of elderly HL has prompted investigations of BV both as monotherapy and in combination for frontline treatment.

- BV + AVD: The phase III ECHELON-1 [29] trial established BV + AVD as an effective frontline regimen in advanced-stage HL. Although the trial predominantly enrolled younger patients, it did include 186 individuals aged 60 or older comprising 14% of the cohort. In the overall population, BV + AVD significantly improved modified PFS compared with ABVD. In the older subgroup, the PFS of BV + AVD was similar to that of ABVD (5-year PFS ~ 67% vs 62%, *P* = 0.44), the subgroup analysis was underpowered to detect differences. By eliminating bleomycin, however, BV + AVD reduced pulmonary toxicity, with any-grade pulmonary events occurring in 2% of patients compared to 13% with ABVD​. The trade-off was an increase in neuropathy and neutropenia: grade ≥ 3 peripheral neuropathy occurred in 18% of patients receiving BV + AVD compared with 3% with ABVD, and any-grade febrile neutropenia was reported in 37% versus 17%, respectively. This underscores that BV is not without toxicity; neuropathy remains a concern, particularly in older patients who may have pre-existing conditions—such as diabetes mellitus, vitamin deficiencies, or infections—that can exacerbate peripheral neuropathy. Nevertheless, BV-induced neuropathy is largely reversible, and neutropenia can be managed with growth factor prophylaxis. This led to BV + AVD, with primary G-CSF support, as the frontline regimen for fit older advanced-stage HL patients. However, its tolerability in older patients warrants careful monitoring.
- Sequential BV and chemotherapy: An approach involving initial BV monotherapy, followed by abbreviated chemotherapy and subsequent BV consolidation, has been demonstrated in older HL patients [12]. By reducing tumor burden upfront without immediate chemotherapy-related toxicity, this strategy permits subsequent treatment with a less intensive regimen. Patients over 60 years received two doses of BV followed by six cycles of AVD and BV consolidation, achieving a 93% complete remission rate and a 2-year PFS of 84%, with only 8% discontinuing treatment and 4% experiencing grade 3 neuropathy. GA assessments showed that moderately fit patients benefited most, while those with high frailty scores experienced higher failure rates.
- BV monotherapy frontline: Given its single-agent activity, BV has been evaluated as the initial treatment for patients unfit for chemotherapy. In a phase II study of 27 patients aged ≥ 60 years treated with up to 16 cycles of BV monotherapy, the ORR was 92%, including a 73% complete remission rate; however, most patients eventually relapsed, with a median PFS of 10.5 months [30]. Similarly, the UK BREVITY trial in frail elderly patients reported a complete remission rate of only 26% after four cycles and a median PFS of 7.3 months [31]. These findings indicate that while BV monotherapy is initially effective, it rarely produces durable remissions in the frontline setting, and most patients ultimately require further treatment. BV can serve as a bridging or palliative option. For instance, frail older patients may receive several cycles of BV to achieve disease control and improve performance status, allowing for a transition to chemotherapy or combined-modality therapy if they become fit enough. Additionally, in cases of localized HL where patients are unable to tolerate chemotherapy or radiation, BV monotherapy may offer temporary disease control.
- BV-based regimens for frail patients: To improve tolerability in frail patients, BV-based regimens have been explored in combination with minimal or attenuated chemotherapy. However, the combination of BV and bendamustine resulted in excessive toxicity, with serious adverse events occurring in 65% of patients, likely due to overlapping myelosuppressive effects [32]. The combination of BV and dacarbazine achieved a 62% complete remission rate and a median PFS of about 18 months, but over 50% of patients discontinued therapy because of adverse effects such as neuropathy and hematologic toxicity [32]. These findings indicate that even low-intensity chemotherapy can substantially increase toxicity when combined with BV in frail individuals. Therefore, BV-based combination regimens should be used with caution—ideally within a clinical trial setting—and require comprehensive geriatric assessment and close toxicity monitoring.

Peripheral neuropathy is the dose-limiting toxicity of BV, particularly in combination regimens, and is more pronounced in older patients. In sequential BV → AVD regimens, limiting exposure to two induction doses plus four consolidation doses resulted in only 4% grade 3 neuropathy, whereas six cycles of BV in the BV + AVD regimen were associated with a higher rate of neuropathy, primarily grade 2. Clinicians may need to reduce or delay the subsequent BV doses if significant neuropathy develops. Overall, BV-related toxicity is manageable with dose adjustments and lacks severe pulmonary or cardiac toxicities commonly seen with conventional chemotherapy, making it a valuable option for older patients with HL.

### Immune checkpoint inhibitors

Classic HL frequently overexpresses PD-L1/PD-L2 due to 9p24.1 alterations, making it sensitive to PD-1 blockade [33]. In EBV-associated cHL, 9p24.1 amplification and PD-L1 expression are further upregulated [34]. Given that EBV is present in approximately 40% of cHL cases, PD-1 blockade offer additional therapeutic benefit in this subset [35]. Anti-PD-1 monoclonal antibodies nivolumab and pembrolizumab yield response rates of approximately 65–80% in relapsed HL, often leading to prolonged remissions. Their side effects—primarily immune-related events such as rash, thyroid dysfunction, and pneumonitis—differ markedly from the myelosuppression, neuropathy, and cardiac toxicity seen with chemotherapy. This non-overlapping toxicity profile suggests that checkpoint inhibitors could be particularly beneficial for older patients, either by reducing the need for chemotherapy or serving as an alternative for frail individuals.

- Nivolumab monotherapy: In a French trial of patients older than 60 years with significant comorbidities (CIRS-G ≥ 6), frontline nivolumab monotherapy resulted in a 28.6% complete metabolic response and a median PFS of 9.8 months [36]. While some patients achieved several months of disease control, most eventually progressed, and 23% died during treatment (2 from toxicity and 6 from lymphoma progression). These findings suggest that while nivolumab may provide temporary clinical benefit for frail patients ineligible for chemotherapy, it is not curative in the frontline treatment of older HL.
- BV + Nivolumab: Combining BV and nivolumab leverages their non-overlapping toxicities to create a chemotherapy-free regimen. In younger relapsed HL patients, this combination has achieved approximately 85% ORR and even cured some without chemotherapy [37]. However, in the phase II trial evaluating frontline treatment for patients aged ≥ 60, the regimen achieved an ORR of 64%, falling short of the predefined target. Complete metabolic remission was observed in 52% of patients, with a median PFS of approximately 18.3 months—or not reached among those who achieved complete metabolic response [38]. Notably, around 48% of patients experienced peripheral neuropathy, and there was one sudden cardiac death potentially related to treatment. Although the efficacy did not meet expectations for all patients, BV combined with nivolumab induced durable remissions in a subset of frail patients. These findings suggest the potential for further optimization—such as incorporating low-dose chemotherapy or using the regimen in an induction setting—while also highlighting the need for caution due to the observed toxicity profile.
- Checkpoint inhibitors combined with chemotherapy: While the ECHELON-1 trial previously demonstrated improved PFS and OS with BV + AVD over ABVD in the general population, the benefit did not extend to patients aged ≥ 60 years [39]. In this subgroup, PFS and OS were comparable between BV + AVD and ABVD, and BV + AVD was associated with significantly higher rates of peripheral neuropathy (any grade: 65%; grade ≥ 3: 18%) and febrile neutropenia (37%)—highlighting the limitations of BV-based therapy in older adults. This underscores the need for alternative frontline strategies in older patients that maintain efficacy while minimizing toxicity. In this context, the combination of checkpoint inhibitors with chemotherapy offers a promising approach by potentially enhancing antitumor activity through immune modulation, while avoiding the neurotoxicity commonly associated with BV-based regimens.
- Nivolumab + AVD (Nivo + AVD): The phase III S1826 trial compared Nivo + AVD with BV + AVD in adults with newly diagnosed advanced-stage classical HL and showed a significant improvement in outcomes. Among patients aged ≥ 60 years (approximately 10% of the cohort), Nivo + AVD achieved a 1-year PFS of 93% versus 64% with BV + AVD, and a 1-year OS of 95% versus 83% [40]. Nivo + AVD also demonstrated a more favorable toxicity profile. Peripheral sensory neuropathy occurred in 32% of patients receiving Nivo + AVD compared with 66% with BV-AVD. Grade ≥ 2 events were reported in 10% and 49%, respectively, and grade 3 events in 2% and 11%. Peripheral motor neuropathy was less frequent with Nivo + AVD (8%, all grade; none grade ≥ 2) compared with BV + AVD (15%, all grade; 8% grade ≥ 2). Non-relapse mortality was also lower with Nivo + AVD (4% vs 14%). While grade ≥ 3 neutropenia was more frequent with Nivo + AVD (48% vs 30%), serious infections were less common (6% vs 21%). Notably, G-CSF prophylaxis was required in the BV + AVD arm but used in only 69% of patients receiving Nivo + AVD, underscoring the regimen's relative safety. Immune-related adverse events—such as hypothyroidism (15%) and rash (11%)—were mostly low-grade and manageable, with no excess pulmonary, hepatic, or gastrointestinal toxicity observed. Collectively, these results establish Nivo + AVD as a highly active and better-tolerated frontline option for fit older patients with advanced-stage HL. Although the trial did not include formal geriatric assessments and the older subgroup was relatively small, the demonstrated efficacy and safety profile support Nivo + AVD as a new standard of care in this population. Future studies incorporating fitness-adapted strategies will be essential to expand its use to more vulnerable elderly patients.
- Pembrolizumab followed by AVD: A phase II multicenter trial explored the efficacy and safety of sequential pembrolizumab followed by AVD chemotherapy in newly diagnosed patients with early unfavorable or advanced-stage HL [41]. Thirty patients, including 4 over age 60, received 3 doses of pembrolizumab, followed by 4–6 cycles of AVD. Complete metabolic response was achieved in 37% after pembrolizumab alone and in 100% after two cycles of AVD. Importantly, no patients experienced progression, death, or required further therapy, with a median follow-up of 22.5 months. The regimen was well tolerated; only two patients developed reversible grade 3–4 immune-related events (transaminitis and Bell’s palsy), and no treatment discontinuations occurred. Radiotherapy was omitted even in bulky disease. Although older patients were underrepresented and no geriatric assessment was conducted, the combination was effective across subgroups. This sequential strategy offers a chemotherapy-sparing, immunotherapy-driven alternative warranting further study in older HL populations.
- Emerging PD-1/PD-L1 Blockade Options: In addition to approved PD-1 inhibitors (nivolumab and pembrolizumab) for cHL, several novel agents have been developed, expanding treatment options for R/R patients. Sintilimab and tislelizumab, both humanized IgG4 anti-PD-1 monoclonal antibodies approved in China, demonstrated high efficacy in phase II trials involving heavily pretreated R/R cHL patients. In the ORIENT-1 trial, sintilimab achieved an ORR of 80.4% and CR rate of 34.3% [42]. Similarly, tislelizumab yielded an ORR of 87.1% and CR rate of 62.9%, with manageable toxicity profiles [43]. Zimberelimab, a fully human IgG4 PD-1 antibody, showed an ORR of 90.6% (CR 32.9%) in a phase II study of 85 R/R cHL patients, with acceptable safety [44]. Avelumab, a human IgG1 anti-PD-1 antibody with antibody-dependent cellular cytotoxicity potential, was evaluated in the JAVELIN phase I trial, showing modest efficacy (ORR 41.9%, CR 19.4%) in 31 heavily pretreated patients [45]. Among these, only tislelizumab and avelumab trials included older adults. In the tislelizumab study, four patients (5.7%) were aged ≥ 65 years, showing consistent efficacy with low-grade hematologic toxicity [44]. In the JAVELIN trial, seven patients (22.6%) were ≥ 65 years, with one case of grade 4 thrombocytopenia [44, 45]. These data support the potential feasibility of novel PD-1 inhibitors in older R/R cHL patients. However, evidence remains limited, and further studies are needed to validate their efficacy and safety, particularly in elderly and frontline settings.

In summary, checkpoint inhibitors offer promising options for older patients with HL by reducing the need for intensive chemotherapy while preserving efficacy, particularly when used in combination regimens (Table 1). Although immune-related adverse events can occur, combining checkpoint inhibitors with chemotherapy may help mitigate these effects. As monotherapy, their use is likely limited to palliative care in very frail patients due to limited curative potential. Ongoing trials are evaluating their integration into first-line regimens across all age groups, with potential applicability to fit older adults under careful monitoring. In the relapsed setting, where autologous stem cell transplant is often not feasible, nivolumab or pembrolizumab have become standard therapies, offering durable disease control with acceptable toxicity.

**Table 1:** Selected upfront treatment strategies in older patients with Hodgkin lymphoma

Regimen	Patient Population (Eligibility)	Key Outcomes (Response/Survival)	Notable Toxicity Findings
**ABVD (doxorubicin, bleomycin, vinblastine, dacarbazine)​ ** [6]	Fit older patients. Advanced-stage HL ≥ 60	~ 80–90% overall response; 5-year OS ~ 65% in ≥ 60 (vs ~ 90% if < 60)​. In early-stage, OS ~ 78% when combined with RT​	High bleomycin lung toxicity (12–25% incidence in > 60)​; ~ 18% treatment-related death in ≥ 60 on trials​. Severe infection in 15%​
**BV + AVD (ECHELON-1 ≥ 60 subset) ** [29]	Advanced-stage HL patients ≥ 60 (14% of trial population). Fit for intensive therapy	5-year PFS 67.1% with BV + AVD vs 61.6% with ABVD (HR 0.82, *P* = 0.44) – no significant difference in older subgroup. ORR ~ 94% in both arms	Pulmonary toxicity: 2% (BV + AVD) vs 13% (ABVD)​. Grade 3–4 neuropathy: 18% vs 3%. Febrile neutropenia: 37% vs 17%​ (BV + AVD arm required G-CSF support)
**Sequential BV → AVD ** [12]	Phase II in patients ≥ 60 (including some with comorbidities, median CIRS score 7). Geriatric-fit and unfit included	2-year PFS 84% and OS 93%​. Patients with high comorbidity (CIRS ≥ 10) had 2-yr PFS only 45% vs 100% if CIRS < 10​, reflecting impact of frailty	Generally well tolerated. Grade ≥ 3 neuropathy 4%​. Allowed delivery of full-dose AVD in most. Approach deemed effective and tolerable for fit older patients
**PVAG (prednisone, vinblastine, doxorubicin, gemcitabine) ** [26]	Phase II in patients 60 to 75 with intact organ function and good general condition (WHO index ≤ 2) with early unfavorable or advanced stage (per GHSG risk stratification)	3-year OS and PFS 66% and 58%, respectively. For advanced stage patients, 3-year OS and PFS were 64% and 56%, respectively	Feasible: 64% completed per protocol and 79% maintained relative dose intensity ≥ 80%. Frequent but manageable toxicity: grade 3–4 toxicity 75% —mostly leukopenia (53%) and infection (23%). Pulmonary toxicity: 7% (1 spontaneously resolving dyspnea, 2 pneumonia, 1 gemcitabine-induced pneumonitis). TRM 1.7%. Limitation due to absence of GA
**BV + Dacarbazine ** [46]	Frail patients ineligible for multi-agent chemo (per GA). Median age ~ 75	Median PFS 17.9 months​. Some durable remissions were achieved, but most patients relapsed by 2 years	Considerable toxicity (> 50% patients had to stop therapy early due to adverse events)​. No treatment-related deaths occurred, but cumulative neuropathy and cytopenias were significant
**BV Monotherapy ** [31]	Frail patients ≥ 60 (co-morbid). Frontline single-agent BV given for up to 16 cycles or until progression	Median PFS ~ 10–11 months​. In the BREVITY trial, CMR after 4 cycles was only 25.8%, and median PFS 7.3 months​. Median OS ~ 20 months	BV alone was well tolerated short-term: peripheral neuropathy ~ 30% (mostly grade 2)​. Fatigue and neutropenia were present in some. Toxicity led to some dose holds but was generally manageable. Lacks long-term disease control —considered suboptimal as sole therapy
**Nivo + AVD (S1826 ≥ 60 subgroup) ** [15]	Advanced-stage HL patients ≥ 60 (10% of enrolled patients). Fit for intensive therapy	1-yr PFS 93% vs 64% (BV + AVD); 1-yr OS 99% vs 98%	Peripheral sensory neuropathy (grade ≥ 2: 10% vs 49%, grade ≥ 3: 2% vs 11%), motor neuropathy (all grade: 8% vs 15%, grade ≥ 2: 0% vs 8%). Grade ≥ 3 neutropenia 49%, but lower rates of serious infections (6% vs 21%) than BV + AVD. G-CSF used in 69% vs 100%. Immune-related AEs: hypothyroidism 15%, rash 11% (mostly low-grade). Fewer treatment discontinuations (15% vs 39%). Overall, more favorable toxicity profile than BV + AVD

*Abbreviations: AVD* doxorubicin, bleomycin, vinblastine, dacarbazine, *BV* Brentuximab Vedotin, *PVAG* prednisone, vinblastine, doxorubicin, gemcitabine, *PFS* progression-free survival, *OS* overall survival, *ORR* overall response rate, *CR* complete response, *CMR* complete metabolic response, *TRM* treatment-related mortality, *GA* geriatric assessment, *FN* febrile neutropenia, *G-CSF* granulocyte colony-stimulating factor, *CIRS* cumulative illness rating scale, WHO index World Health Organization Index, *RT* radiotherapy

### Other Novel/Targeted Therapies

In addition to conventional therapies, several emerging immunotherapies—including immunomodulatory drugs and cell-based approaches—are under investigation for elderly patients with HL.

- Lenalidomide: Lenalidomide is an immunomodulatory drug that can enhance immune response. A phase I trial combined lenalidomide with AVD in patients over 60 [47]​. The regimen achieved an ORR of 86% and a 3-year PFS of approximately 70%, indicating clinical activity; however, substantial hematologic toxicity was observed. The study suggested that AVD may not be an optimal backbone for lenalidomide due to overlapping bone marrow suppression. Future research may better define the role of lenalidomide or similar agents, potentially as part of less intensive regimens or in maintenance settings for older patients with HL.
- Cellular therapies: While cellular therapies including Chimeric Antigen Receptor (CAR) T-cell therapy are transforming the treatment landscape of lymphoid malignancies [48–51], their role in HL remains investigational. Considering that cHL is characterized by a highly immunosuppressive tumor microenvironment (TME) and diminished HLA expression on HRS cells, adoptive T-cell therapy may offer a means to circumvent these mechanisms of immune evasion [52].
  - CD30 CAR T-cell therapy: CD30 is highly expressed on HRS cells and minimally on normal tissues, making it an attractive target for CAR T-cell therapy in cHL. Early-phase trials of CD30 CAR T cells have demonstrated manageable toxicity and encouraging clinical activity in R/R cHL, including older patients and those heavily pretreated with BV or checkpoint inhibitors. Fludarabine-based lymphodepletion appears to enhance CAR T-cell persistence and response rates. Across trials, ORRs range from 29% without lymphodepletion to 62% with lymphodepletion, with CRs ranging from 29 to 51%, respectively. Notably, Cytokine release syndrome (CRS) has generally been mild, no cases of immune effector cell-associated neurotoxicity syndrome (ICANS) were reported, and significant infectious complications have not been observed. Grade 3–4 cytopenias occurred primarily in studies using lymphodepleting regimens. Investigational strategies include CCR4 co-expression to enhance tumor trafficking, allogeneic CD30 CAR-EBV-specific T-cells, and sequential use of immune checkpoint inhibitors [52–54]. Given the intensity of treatment, CD30 CAR T-cell therapy may be best suited for carefully selected older patients who are able to tolerate lymphodepleting chemotherapy; however, its safety and efficacy in elderly populations warrant further prospective validation.
  - EBV-specific T-cell therapy: cHL is generally characterized by a sparse population of malignant HRS cells embedded within an abundant, immune cell-rich microenvironment. The distinct histological profile of cHL provides a strong rationale for immunotherapeutic strategies targeting EBV antigens, given that approximately 40% of cases are EBV-positive—offering a unique opportunity for EBV-specific T-cell (EBVST) therapy. HRS cells exhibit a type II latency pattern characterized by latent membrane proteins 1 and 2 (LMP1 and LMP2), EBV nuclear antigen 1 (EBNA1), and *Bam*H1-A right frame 1 (BARF1) expression. Therefore, the development of LMP-specific cytotoxic T lymphocytes (CTLs) has demonstrated clinical efficacy in cHL, achieving a CR rate of 96% in patients in remission and 52% in those with active disease [52, 55]. Furthermore, engineering EBVSTs to express a dominant-negative TGF-β receptor has shown to enhance efficacy by counteracting the immunosuppressive TME [52, 56]. Allogeneic EBVSTs have also proven to be feasible and effective for both remission and active disease following transplantation [52, 57]. Recent strategies have focused on co-expressing CD30-directed CARs on EBVSTs (CD30.CAR-EBVSTs) to increase tumor specificity and persistence. The ongoing BESTA trial is investigating allogeneic CD30.CAR-EBVSTs in patients with R/R CD30-positive lymphomas, including elderly patients with cHL [53]. However, the use of allogeneic EBVSTs raises concerns regarding graft-versus-host disease, which necessitates further evaluation in clinical trials. Despite these challenges, off-the-shelf allogeneic EBVSTs derived from healthy donors represent a promising, scalable, and potentially low-toxicity immunotherapeutic approach for elderly patients with EBV-positive cHL, particularly those with impaired autologous T-cell function due to immunosenescence or prior therapies. Ongoing studies will be essential to establish their long-term safety and efficacy in this population, including the understudied role of CD4^+^ T cells in sustaining antitumor immunity [51, 52, 57, 58].

While effective, targeted agents are associated with substantial financial costs. Immune checkpoint inhibitors, in particular, can lead to immune-related toxicities that may necessitate prompt immunosuppressive treatment. Older patients are especially vulnerable, as corticosteroids used to manage these toxicities can cause significant side effects, underscoring the importance of vigilant supportive care. Despite these challenges, CD30- and PD-1–targeted therapies have improved outcomes in older HL patients. BV has benefited patients intolerant to bleomycin, and checkpoint inhibitors may reduce reliance on cytotoxic agents. Future frontline regimens may incorporate targeted therapies with minimal chemotherapy—e.g., PD-1 inhibitors plus BV, followed by abbreviated AVD and possible anti-PD-1 maintenance—to optimize efficacy while reducing toxicity. In fit older patients with advanced-stage disease, Nivo + AVD is expected to become the new standard of care.

## Supportive care strategies for older HL patients

Optimal management of the patients with older HL requires comprehensive supportive care to enhance treatment tolerance and quality of life. Key strategies include:

- Pre-Phase Therapy: The majority of studies on pre-phase treatment have been conducted in aggressive non-Hodgkin lymphomas [15]. In particular, the use of pre-phase steroids is widely recommended for elderly, treatment-naïve patients with DLBCL. In the German High-Grade Non-Hodgkin Lymphoma Study Group (DSHNHL) trials, which included elderly DLBCL patients aged 61 to 75 years, pre-phase treatment with a single intravenous injection of vincristine (1 mg) and oral prednisone (100 mg daily for 5–7 days) was shown to reduce the incidence of tumor lysis syndrome, improve functional status, and decrease treatment-related mortality, particularly during the first cycle. Notably, similar beneficial effects were also observed with prednisone alone [59, 60]. Although further studies are warranted to determine the efficacy, optimal dosage, and duration of pre-phase steroids in older patients with cHL, expert guidelines currently recommend the use of pre-phase steroids in this population (e.g., oral prednisone 60–80 mg daily for 5 days) [15].
- Growth Factor Support: Older patients are particularly susceptible to chemotherapy-induced neutropenia and its complications due to diminished marrow reserve. In the ECHELON-1 trial [29], primary prophylactic G-CSF—including 34% long-acting and 73% short-acting formulations—reduced rates of grade ≥ 3 neutropenia (70% vs 29%), febrile neutropenia (21% vs 11%), and hospitalization (38% vs 29%) in patients receiving BV + AVD. G-CSF prophylaxis also minimized treatment delays across all agents—BV (49% vs 35%), doxorubicin (50% vs 37%), vinblastine (50% vs 35%), and dacarbazine (49% vs 37%)—thereby helping to preserve dose intensity. Importantly, G-CSF prophylaxis did not increase pulmonary toxicity, which was a concern in BV-based regimens [61]. While these findings support routine G-CSF prophylaxis use, the study was not elderly-specific and included a limited G-CSF prophylaxis subgroup, highlighting the need for further data in older populations.
- Infection Prophylaxis and Monitoring: Older patients face an elevated risk of infection [6]. Prophylactic strategies may include trimethoprim-sulfamethoxazole (TMP-SMX) (particularly during prolonged corticosteroid use), age-appropriate vaccinations (e.g., influenza, COVID-19, shingles), and, in selected cases, prophylactic fluoroquinolones. During febrile episodes, prompt evaluation with a low threshold for hospitalization and initiation of intravenous antibiotics is essential to mitigate severe infectious complications.
- Organ Function Surveillance:
  o Pulmonary: Baseline pulmonary function tests including diffusing capacity for carbon monoxide (DLCO) and vigilant screening (e.g., pulse oximetry) are recommended to detect bleomycin toxicity, with early discontinuation if necessary. Early discontinuation is advised if toxicity emerges—especially given that a BC Cancer study found that 38% of lung toxicity cases occurred within the first two cycles [2].
  o Cardiac: Baseline echocardiogram or multigated acquisition scan is recommended before anthracycline use, with cardioprotective strategies (dexrazoxane, liposomal doxorubicin) for at-risk patients [62].
  o Neurotoxicity: Baseline and regular neurologic assessments help detect and manage neuropathy, with dose adjustments as necessary. Validated peripheral neuropathy grading tools should be considered to guide BV dose modification in elderly HL patients.
  o Renal: Regular monitoring of creatinine and electrolytes, with emphasis on maintaining hydration.
- Dose Adjustments and Schedule Modifications: Adjusting the dose or extending the treatment interval—for example, modifying the schedule from every 2 weeks to every 3 weeks—can help mitigate toxicities such as neutropenia or neuropathy. This approach allows for the continuation of therapy at a reduced intensity, minimizing the need for treatment discontinuation.
- Psychosocial Support and Rehabilitation: Engaging social workers, nutritionists, and physical therapists can help address challenges such as social isolation, nutritional deficiencies, physical deconditioning, cognitive changes, and mood disorders. Early involvement of palliative care supports effective symptom management and facilitates goals-of-care discussions. Additionally, studies have shown that maintaining physical activity during treatment improves clinical outcomes and reduces fatigue [63].

## Outlook

Outcomes for older patients with HL have remained suboptimal, with cure rates still lagging behind those of younger individuals. However, recent advances such as the integration of brentuximab vedotin and the emergence of Nivo + AVD have improved efficacy and tolerability in fit older adults. These benefits, however, have not fully extended to frail patients, who continue to face high risks of relapse and treatment-related toxicity. Chemo-free regimens involving BV or checkpoint inhibitors may offer temporary disease control but rarely induce durable remissions in the frontline setting. Improving outcomes across the full spectrum of older HL patients will require treatment strategies that are individualized based on geriatric assessment, incorporate novel agents in less toxic regimens, and are promoted by robust supportive care to mitigate complications and preserve function. Advances in biomarker-driven risk stratification may further refine therapeutic decision-making. Ultimately, narrowing the survival gap between older and younger patients will depend on the development of precision, tolerance-adapted therapies guided by continued translational/clinical research and multidisciplinary care.

## Data Availability

No datasets were generated or analysed during the current study.
